# Melioidosis: an unusual cause of recurrent buttock abscesses

**DOI:** 10.1111/j.1365-2230.2012.04469.x

**Published:** 2013-06

**Authors:** C I Wootton, I A M Elliott, D Sengdetkha, M Vongsouvath, S Phongmany, D A B Dance

**Affiliations:** * Department of Dermatology, Queen's Medical CentreNottingham, UK; † Microbiology Laboratory, Lao-Oxford-Mahosot Hospital Wellcome-Trust Research Unit, Mahosot HospitalVientiane, Lao People's Democratic Republic; ‡ Centre for Clinical Vaccinology and Tropical Medicine, Churchill Hospital, University of OxfordUK; § Adult Infectious Diseases ward, Mahosot HospitalVientiane, Lao People's Democratic Republic E-mail: ciwootton@aol.com

An 18-year-old woman presented to Mahosot Hospital, Vientiane with a 3-year history of widespread abscess formation and significant scarring of her left buttock. The patient was a rice farmer from Saravan Province. The lesion had initially started as a small painless pustule, without preceding trauma or injection. It had grown in size, and had been treated with incision and drainage and a week’s course of ampicillin. However, the lesion persisted, and further abscesses developed despite subsequent (self-initiated) ampicillin therapy and treatment from a traditional Lao healer. The patient remained systemically well throughout with no fever or other symptoms.

**Figure 1:**
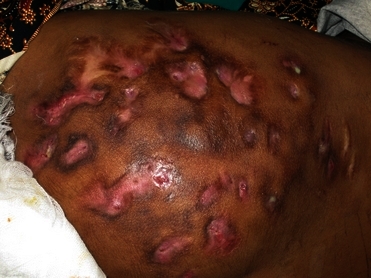
Cutaneous melioid affecting the buttock.

On physical examination, areas of atrophic scarring, induration, and discharging sinuses were seen on the patient’s left buttock (Fig. 1). The remainder of the buttock area was clear, and the rest of the clinical examination was unremarkable.

Blood tests revealed that the patient had microcytic anaemia, with haemoglobin 8.2 g/L (normal range 11.5–16.5 g/L) and mean corpuscular volume 50 fL (80–96 fL). Glucose and creatinine were within normal ranges, and screening for thalassaemia gave negative results.

There was no growth from two sets of blood cultures. A pus culture yielded a pure growth of an oxidase-positive Gram-negative rod with a metallic sheen on blood agar, which was confirmed as *Burkholderia pseudomallei* by a biochemical test kit (API20NE; bioMerieux, Marcy L’Etoile, France).

**Figure 2:**
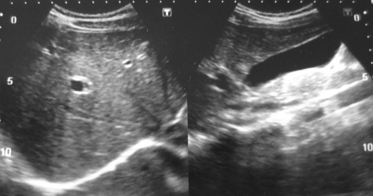
Ultrasound scan of splenic microabcesses.

**Figure 3:**
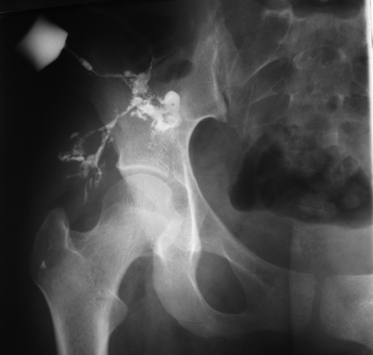
Fistulogram of buttock lesion.

A chest radiograph was normal, but abdominal ultrasonography identified splenic microabscesses (Fig. 2). A fistulogram of the left buttock demonstrated no connection with underlying deep structures (Fig. 3). Ultrasonography of the buttock revealed a collection of pus within the soft tissue, 80 × 10 mm in size, which was drained percutaneously.

A diagnosis of melioidosis was made, and the patient was treated with intravenous ceftazidime (2 g three times daily) for 3 weeks because of the delay in abscess drainage, evidence of splenic involvement and persistently culture-positive pus samples. The patient was discharged on a combination of oral cotrimoxazole (240/1200 mg twice daily) and doxycycline (200 mg once daily) for a further 16 weeks. At 3 months, there was marked improvement, with only one small sinus present without significant pus discharge. A swab of the site was culture-negative.

Melioidosis is the clinical manifestation of infection with *B. pseudomallei*, a ubiquitous saprophytic environmental bacterium that resides in the water and soil of predominantly tropical climates, most notably south-east Asia and northern Australia.^1^ The area from where the patient originated, Saravan Province, is know to have a high prevalence of *B. pseudomallei* in the soil. Typically, infection occurs when working closely with soil, such as in paddy fields. Direct inoculation through breaks in the skin barrier and inhalation are recognized modes of transmission. Systemic disease, with or without dissemination, is typical, but localized cutaneous and soft-tissue disease is well described, and accounts for around 12–24% of clinical presentations of melioidosis.^2^ Skin presentations include subcutaneous abscesses, pustules, wound infections, cellulitis, and granulomatous lesions. Mortality ranges from 20% to 50% in Australia and Thailand, respectively.^3^ Although melioidosis is almost exclusively a disease of tropical climates, sporadic imported cases do occur among both immigrants and adventure tourists.^4^

This case was particularly interesting because of the extensive nature of the disease and long history, resulting in significant scarring that is unlikely to improve with treatment.
